# A New Semantic Segmentation Framework Based on UNet

**DOI:** 10.3390/s23198123

**Published:** 2023-09-27

**Authors:** Leiyang Fu, Shaowen Li

**Affiliations:** 1 School of Information & Computer Science, Anhui Agricultural University, Hefei 230036, China; fly2008@ahau.edu.cn; 2 Anhui Provincial Key Laboratory of Smart Agricultural Technology and Equipment, Hefei 230036, China

**Keywords:** semantic segmentation, UNet, ensemble method, machine vision, environmental perception

## Abstract

This paper discusses a semantic segmentation framework and shows its application in agricultural intelligence, such as providing environmental awareness for agricultural robots to work autonomously and efficiently. We propose an ensemble framework based on the bagging strategy and the UNet network, using RGB and HSV color spaces. We evaluated the framework on our self-built dataset (Maize) and a public dataset (Sugar Beets). Then, we compared it with UNet-based methods (single RGB and single HSV), DeepLab V3+, and SegNet. Experimental results show that our ensemble framework can synthesize the advantages of each color space and obtain the best IoUs (0.8276 and 0.6972) on the datasets (Maize and Sugar Beets), respectively. In addition, including our framework, the UNet-based methods have faster speed and a smaller parameter space than DeepLab V3+ and SegNet, which are more suitable for deployment in resource-constrained environments such as mobile robots.

## 1. Introduction

Machine vision refers to using computers to simulate human visual functions, but it is not just a simple extension of human eyes. It can extract image information for detection, measurement, and control. This paper discusses a semantic segmentation framework in the field of agricultural robots. The agricultural environment and objects are very complex, the efficiency of manual labor is low, and the excessive use of chemical fertilizers and herbicides brings source pollution. Research on semantic segmentation methods can improve the environmental awareness of agricultural robots, which is the prerequisite for robots to work autonomously and efficiently. We aim to perform semantic segmentation of crops under natural light conditions so that robots can freely perceive in the wild. However, the challenges come from (a) harsh outdoor environments (different lighting conditions and changing soil background) and (b) complex biological systems (there are many types of crops and weeds, and there are multiple stages in the crop life cycle).

Image segmentation is a fundamental problem in the field of computer vision. It is the process of dividing an image into multiple sub-regions so that the pixels in each sub-region have similar characteristics. Semantic segmentation is a particular form of image segmentation that divides each pixel in an image into a set of predefined semantic categories independent of object instances. Therefore, semantic segmentation can be viewed as a generalization of image classification problems rather than pixel-level object detection or instance segmentation problems. Semantic segmentation is the basis of many computer vision tasks, such as autonomous driving, intelligent video surveillance, and other fields, because it can help computers understand the semantic meaning of different regions in the image to make more accurate judgments and decisions.

Traditional semantic segmentation methods include clustering algorithms, active contour models, watersheds, random decision forests, support vector machines, etc. The K-means algorithm counts the distance from each sample point to the cluster core and reclassifies iteratively until the cluster core does not change. This algorithm is used in medical image segmentation [1]. The active contour model is an algorithm that segments along the image boundary and expects the contour to be smooth. The algorithm realizes the contour finding by minimizing the energy function. Ref. [2] focuses on brain MRI image segmentation and purification. The watershed algorithm usually divides the grayscale image and interprets the grayscale image as a heat map. The place with a small pixel value is called a watershed basin, and the place with a relatively high value between two watershed basins is called a watershed. The watershed algorithm starts to fill the basin from the minimum value. When the two basins gradually begin to connect to form a watershed, that is, when the algorithm finds the highest point, the segmentation is completed. Ref. [3] provides a detailed description of the derivation and process of the watershed algorithm. The random decision forest algorithm was first proposed in [4], which is essentially a kind of ensemble learning that trains multiple classifiers and synthesizes their classification effects. Support vector machines (SVMs) are another widely studied binary classifier, and the SVMs algorithm was used in the Pascal VOC 2009 and 2010 segmentation challenges in [5].

The algorithm based on deep convolutional neural networks (DCNNs) has become an effective analysis method and the main research direction for semantic segmentation. The hierarchical features obtained by DCNNs can express high-level abstract semantics, and their performance in semantic segmentation tasks has excellent potential. End-to-end deep neural networks have also become a trend in solving semantic segmentation and other problems in computer vision. The fully convolutional deep neural network (fully convolutional neural networks, FCNs) [6] is one of the foundational works that has occupied first place in the Pascal VOC 2012 semantic segmentation competition for a long time. UNet [7] designed semantic segmentation applied to medical images. Due to the particularity of medical image processing, the number of samples available for training is relatively small. The UNet method effectively improves the performance of training and detection using a small number of datasets. Also, it proposes an efficient method for processing large-scale images. SegNet [8] is a deep network for image semantic segmentation proposed by Cambridge to solve autonomous driving and intelligent robots. SegNet is based on FCN and has two versions, SegNet and Bayesian SegNet. The DeepLab series (v1, v2, v3, and v3+) [9,10,11,12] is a series of work performed by Google for semantic segmentation. It is a method that combines deep convolutional neural networks (DCNNs) and probabilistic graphical models (DenseCRFs). There are many other image semantic segmentation methods [13], such as UNet, a network and training strategy that relies on data augmentation to use very few annotated samples more efficiently.

Training deep learning models requires vast datasets. Although some public datasets are available and some methods of synthesizing datasets exist, collecting and labeling datasets are grueling for specific agricultural objects. Data collection can be automated, but manual annotation is time-consuming and inefficient unless the amount of annotation is tiny. Automatic annotation technology has always been a hot spot in research [14], but there is a paradox in generating ground truth. If automatic annotation is implemented, the original problem will no longer exist. For example, if semantic annotation can be completed by simply distinguishing color features, then this type of problem itself can be easily solved. Therefore, we hope to find a solution that (a) is not demanding on the size of the raw dataset but (b) is efficient in execution. We reviewed the relevant literature and found that UNet achieved outstanding results despite the scarcity of raw medical datasets. We also found some applications of UNet in agriculture, such as [15] applying UNet to coleoptile emergence timing detection, which also integrates SEResNet, InceptionV3, and VGG19. Ref. [16] embedded a channel attention mechanism, but their model structures are relatively complex, and many other semantic segmentation methods use similar integration strategies. Ref. [17] proposed a model to facilitate the timely monitoring of weeds in the farmland, which has many parameters. Therefore, we propose an ensemble framework to obtain better segmentation results with few raw annotated datasets. In addition, we focus on reviewing DeepLab V3+ and SegNet and conduct in-depth comparisons with our UNet-based methods in the later experimental section.

The paper is organized as follows: First, the significance of semantic segmentation is introduced in the context of agricultural robot applications, including problems and challenges. Then, traditional and deep-learning-based semantic segmentation methods are analyzed. We focus on the relevant technologies of the framework in this paper, including the mathematical expression of semantic segmentation, UNet infrastructure, and integration methods. The proposed framework section elaborates on the implementation of integrating UNets. The experiment and discussion section introduces the self-built dataset Maize and elaborates on three aspects: data preprocessing, model training and testing, and assessing results and analyses, comparing it with DeepLab V3+ and SegNet. Moreover, we test on a Sugar Beets public dataset (Supplementary Materials). Finally, a summary and outlook are presented.

## 2. Related Works

### 2.1. Formal Expressions of Semantic Segmentation

Here, we outline [18] formal representations for image (semantic) segmentation and machine (deep) learning models. Let A be a collection of images defined in the domain R^d^ (d ≥ 2), where a_i_∈A is an observed image, and P_1_ … P_n_ be n predicates that detect image features such as edges, smoothness, texture, or color logic. In our study, the environmental perception of agricultural robots can be reduced to a binary classification problem, such as crops and environmental background (including weeds and all other non-crop objects). We usually separate the crop (c, crop) and the background (b, background), a_i_ = c∪b. We can define 2 simple predicate logics (*n* = 2), as shown in Formula (1):

P_1_(c) = {F(x) < α,∀x∈c}, P_2_(b) = {F(x) ≥ α,∀x∈b} (1)

Among them, F(x) is the image feature quantization function, α is the critical value, and the value range is (0, 1).

The core idea of using machine (deep) learning to segment images is to tune a general model to a specific solution by learning from sample data (training data). Compared with traditional segmentation methods to extract features directly, machine (deep) learning methods define several segmentation models, with a general form as shown in Formula (2). *A* is the image set, *G* is the ground truth, and θ is the parameter vector. The learning stage is to choose θ to minimize the loss function *L* that measures the prediction segmentation accuracy. Many loss options exist, such as mean square error loss and binary cross entropy (BCE) loss. According to the source of *G*, it can be divided into three learning methods: supervised, semi-supervised, and unsupervised. In supervised learning, the dataset comes with labeled data, unsupervised (self-supervised) learning needs to extract labeled data from the dataset, and semi-supervised learning has a small amount of labeled data, which needs to be supplemented by obtaining labeled data.

(2) $$\theta^{*}=\arg\;\underset{\theta }{\min}\;L(A,\;G,\;\theta )$$

To progressively extract higher-level features from data, machine (deep) learning models use a multilayer structure called a neural network, where the number of layers is the depth of the model. Common neural network structures include multilayer perceptron (MLP), deep auto-encoder (DAE), and convolutional neural network (CNN). A CNN divides an image into small regions and scans one region at a time to identify and extract features for classifying images. CNN is mainly composed of three layers: (a) a convolutional layer, which analyzes several pixels of the image at a time and extracts the underlying features (edge, color, gradient direction, etc.); (b) nonlinear layer, which activates a function to create a feature mapping, where each feature belongs to the probability of the desired class; and (c) pooling layer or sampling layer, which reduces the number of features and calculations in the network, thereby controlling overfitting.

### 2.2. UNet Infrastructure

**Figure 1:**
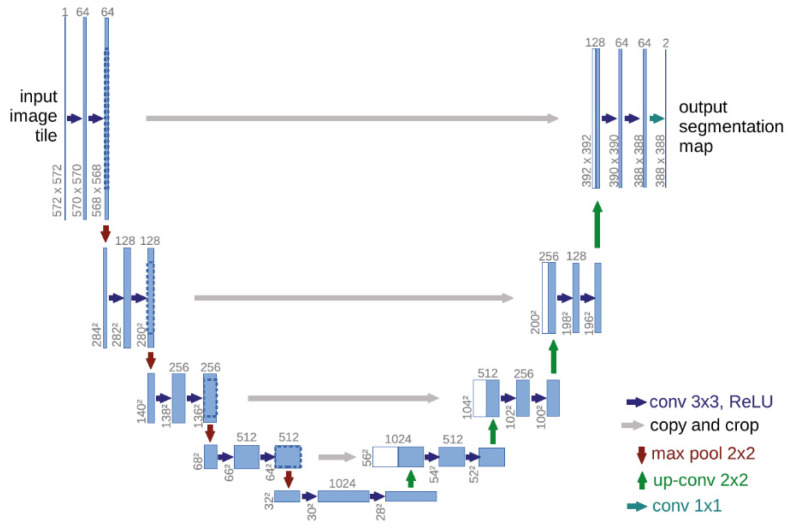
UNet architecture.

While convolutional neural networks excel in many vision tasks, the available training set size and the network size are limiting factors. UNet modified and expanded the architecture of FCN and built an elegant network architecture UNet, which only needs a few raw images and corresponding labels. It expands the training set with the help of data enhancement methods and can produce accurate segmentation results. The main modifications made by UNet to FCN are as follows: (a) Upsampling produces many feature channels, which can propagate contextual information to higher-resolution layers. (b) The expansion path is symmetrical to the contraction path, presenting a U-shape. (c) The network does not have a fully connected layer. The UNet network architecture is shown in Figure 1, including a contraction path (left) and an expansion path (right), with a total of 23 convolutional layers. The shrinkage path follows the typical architecture of convolutional networks, where each downsampling step consists of two 3 × 3 convolutions, each followed by an activation function (ReLU) and a 2 × 2 max pooling operation, doubling the number of feature channels. Each step in the dilation path consists of upsampling the feature map followed by a 2 × 2 convolution (“up convolution”), halving the number of feature channels concatenated with the correspondingly cropped feature map in the shrinkage path. In the last layer, each feature vector is mapped to the desired number of classes using a 1 × 1 convolution.

### 2.3. Ensemble Methods

Researchers traditionally use regular red–green–blue (RGB) images, which help understand visual context descriptions. In addition, researchers have also explored color space models such as HSV [19]. HSV is an abbreviation for hue, saturation, and value. Among them, hue is the primary attribute of color, which is the color that is often identified, such as red, blue, etc. Saturation refers to color. The higher the saturation, the purer and brighter; the lower the saturation, the grayer and darker. The value range of saturation is from 0 to 100%. Brightness is the brightness of the color, and its value ranges from 0 to the maximum value allowed by the computer. Since the value ranges of hue, saturation, and brightness are different, its color space model is represented by a cone. Compared with the shortcomings of the unintuitive connection between the three color components of the RGB model and the final color, the HSV model is more in line with the way humans perceive colors: color, depth, and brightness. It is worth noting that complementary information is provided between color spaces [20,21], and learning this information through aggregation techniques is beneficial.

A single model often has deficiencies, such as UNet segmentation and localization accuracy, which cannot be achieved simultaneously, and the ensemble method uses a linear combination of multiple model prediction results, which, on average, provides better overall accuracy than a single model [22]. Ref. [23] proposed a method called Deep CNN Ensemble, which integrated the two models of HybridSN and ResNet, but the running time of this method increased exponentially due to training multiple models on the same pixel. Ref. [24] proposed an ensemble method called EECNN, which uses random sampling technology in the feature space to obtain a data subset of each sub-model, and some sub-models may reduce the classification accuracy due to the small number of training pixels. Ensemble methods usually have three implementation strategies: bagging, boosting, and stacking [25]. (a) Bagging extracts a subset of the dataset to train sub-classifiers. Each sub-classifier and subset are independent of each other and thus parallelized. (b) Boosting is proposed by the famous AdaBoost algorithm, which uses a complete dataset to train each sub-classifier and then adjusts the weight of the entire integrated network after each iteration. (c) The sub-classifiers of stacking are not parallel networks but are stacked on top of each other linearly. The output of one sub-classifier is used as the input of another sub-classifier. In comparison, boosting and stacking can be very expensive regarding runtime, while bagging can improve accuracy while reducing runtime.

Generally, assuming that the ensemble method has k classifiers [*F*_1_(*x*), …, *F_k_*(*x*)] and the corresponding weight vectors [*ω*_1_, …, *ω_k_*] and that the sum of all weights is 1, Formula (2) can be further expressed as Formula (3):

(3) $$\theta^{*}=\arg\;\underset{\theta ,k,\omega }{\min}\sum_{i=1}^{k}\omega_{i}L(F_{i}(x),y,\theta_{i})$$

where (*x*, *y*) is (pixel, class), derived from the aforementioned *A* image collection and *G* ground truth. For the bagging strategy, *A* and *G* can be divided into k subsets [*A*_1_, …, *A_k_*] and [*G*_1_, …, *G_k_*], and then Formula (3) can be further expressed as Formula (4):

(4) $$\theta^{*}=\arg\;\underset{\theta ,k,\omega }{\min}\sum_{i=1}^{k}\omega_{i}L(F_{i}(A_{i}),G_{i},\theta_{i})$$

Among them, the classifier *F* can choose different or similar models. For example, UNet is used in this paper.

In the ensemble method, there are usually cases where the contribution of some classifiers is more efficient than others. The weight vector [*ω*_1_, …, *ω_k_*] is an essential parameter for adopting the results of classifiers, which can increase the ratios of classifiers with more contributions. Weights can be valued in three ways. (a) Constant weights: Each model is given equal attention by multiplying the loss of each model by a constant. (b) Abundance weights: The weights for each model loss equal the pixel abundance percentage. For example, if 10% of the data goes into a model, the weight is set to 0.1. (c) Random weights: as long as the sum of the weights equals 1, the weights are randomly assigned.

## 3. Materials and Methods

### 3.1. Self-Build Dataset Maize and Robot Platform

**Figure 2:**
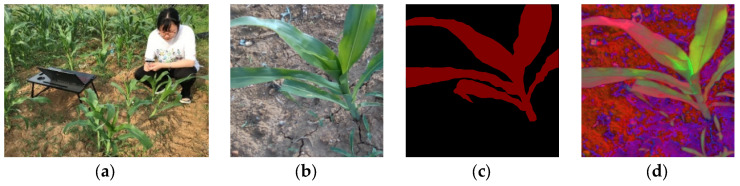
Maize data collection and processing. From left to right: (**a**) data collection; (**b**) RGB; (**c**) mask; (**d**) HSV.

To test the effectiveness of the framework in this paper, which is also an essential part of the environmental perception of agricultural robots, we selected corn plants in the agricultural extract garden of Anhui Agricultural University as the semantic segmentation object to provide information for subsequent robot operations (such as weed removal). We collected pictures and videos of corn (including background and weeds) (Figure 2a). The acquisition device is Intel^®^ RealSense™ Depth Camera D435i (Intel, Santa Clara, CA, USA). The main parameters are depth resolution and an FPS of 1280 × 720 and 30 fps. The camera can simultaneously record the depth information to further measure the distance from the robot arm (camera) to the corn.

**Figure 3:**
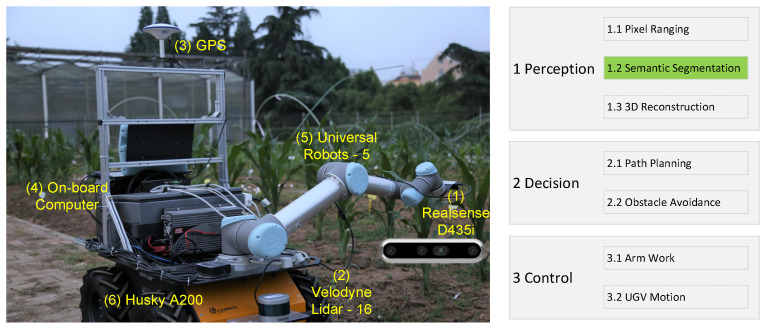
Our robot platform. (**Left**): the robot entity; (**Right**): a three-tier architecture. Labeled green 1.2 is our model.

Figure 3 shows our robot platform. It can be defined as a multi-tier system, and typically, we can consider three tiers: (a) perception, (b) decision, and (c) control. This architecture allows us to define a simple and universal method for all solutions. Furthermore, it has enough flexibility to consider any given environment. Our framework can run on an onboard computer, with real-time images captured by a Realsense D435i camera. Integrating more models and fast sensing methods for new agricultural operation objects is still valuable for further research.

### 3.2. Proposed Framework

**Figure 4:**
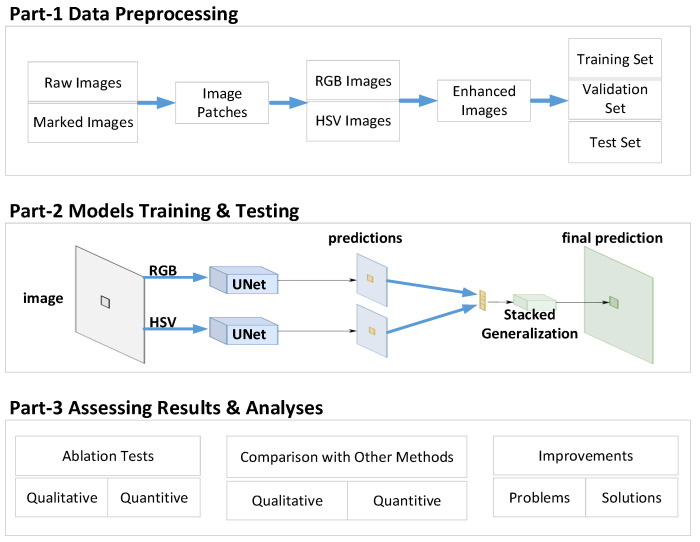
Structure of the lightweight crop semantic segmentation framework.

This paper aims to create an ensemble framework based on the bagging strategy and UNet network to improve crop segmentation accuracy and reduce operational complexity. A significant problem with aggregation-based techniques is the increased computational overhead, and our goal is to design a lightweight semantic segmentation network (Figure 4) that takes advantage of aggregation techniques while considering computational overhead. This work tackles a crop semantic segmentation problem to provide perception capabilities for agricultural robotic operations. We only study the semantic segmentation of binary classification. Each pixel in the dataset image belongs to only one of 2 classes/labels, namely crop or background. First, we convert the raw RGB image to an HSV color space. Then, we feed the raw and transformed images into our ensemble UNet networks to obtain segmentation masks. Then, we perform pixel-wise addition to obtain the final prediction mask. Finally, the corresponding predicted classes are obtained.

The main contributions of this paper are as follows:

(a) We proposed an ensemble framework based on the bagging strategy and the UNet network, using RGB and HSV color spaces to improve the segmentation effect.
(b) We evaluated our framework on the self-built dataset Maize and a public dataset Sugar Beets. We compared it with UNet-based methods (single RGB and single HSV), DeepLab V3+, and SegNet. Our framework has advantages in terms of parameter size and execution speed.

As can be seen from Figure 4, our framework can be divided into three parts: data preprocessing, model training and testing, and result evaluation and analysis, which are described in detail below.

(a) Data preprocessing: This part’s primary work is preparing datasets. In this paper, the pictures of corn plants are self-built (see the experiment and discussion section for details), and the real-value pictures are manually annotated. To simplify the calculation and operation, the size of the picture is adjusted uniformly, and the RGB image is converted into an HSV image. The geometric transformation methods mainly include flip, rotation, cropping, scaling, translation, etc. To prevent overfitting, pixel transformation methods such as adding salt and pepper noise, Gaussian blur, etc., can also be used. Finally, it is divided into training, verification, and test subsets according to a specific ratio.
(b) Model training and testing: The main work of this part is to train the model. Since raw image pixel values range from 0 to 255, high numerical pixel values require more computing power. Therefore, we follow the image normalization process to normalize the pixel values to the 0 to 1 range by dividing each pixel value by the highest possible pixel value (i.e., 255). We introduce a framework composed of different color spaces to offset the influence of lighting conditions. The RGB and HSV color spaces can generate complementary information, and our framework can capture and aggregate it to obtain more discriminative information. Our proposed framework is to train UNet-based models (single RGB and single HSV) to generate different predictions. Then, the classical stacked generalization [26] is applied to obtain the final prediction, the crop, and the background (all other elements are classified to none crop). Algorithm 1 describes the framework in this paper formally.
(c) Assess and analyze the results: The main work of this part is framework evaluation. We carry out the ablation experiment of the framework in this paper and the experiment of similar methods and make a horizontal comparison from two qualitative and quantitative perspectives. The advantages and disadvantages of the framework in this paper are analyzed, and further improvements are proposed.

**Algorithm 1:** Ensemble UNetsInput: *A*, the set of images, and *G*, the ground truth. Output: *a_i_* = *c*∪*b*, *a_i_*∈*A*, *c*: crop, *b*: background, loss, val_loss, accuracy.Init k, the model number, and n, the epoch size, and a weight vector [*ω*_1_, …, *ω_k_*] Prepare raw image dataset [*A*_1_, …, *A_k_*] and marked image dataset [*G*_1_, …, *G_k_*] Init *LO*, *AC* for *j* = 0, …, *n*    Init *LO_j_*, *AC_j_*    for *i* = 0, …, *k*     Train *F_i_*, feed with *A_k_* and *G_k_*     Get lost value *LO_ji_* and accuracy *AC_ji_*     Sum *LO_j_* + = *ω_i_LO_ji_*, *AC_j_* + = *AC_ji_*    end for    Sum *LO* + = *LO_j_*, *AC* + = *AC_j_* end for Get average lost *LO*/*n*, and average accuracy *AC*/*n* Stacking [*F*_1_, …, *F_k_*] predictions to get the final prediction.

## 4. Experiment and Discussion

### 4.1. Data Preprocessing

Just like UNet, whose training data is a set of 30 images (512 × 512 pixels) from serial section transmission electron microscopy of the Drosophila first instar larva ventral nerve cord (VNC), we only needed a tiny raw dataset, and the images and videos collected in our field were separated into 30 images of individual corn, which was very meaningful for alleviating the burden of data collection. The image annotation tool Lableme created the semantic annotation map corresponding to the plant image, using the function named cvtColor() of the open computer vision library Opencv2 to convert plant images into HSV format images. The RGB and HSV images shared the same semantic annotation map. We used the ImageDataGenerator class of the artificial neural network library Keras to achieve image enhancement (rotation, movement, scaling, etc.). It should be noted that the semantic annotation map and the raw image are converted synchronously. Finally, we brought the amount of the total dataset to 4000 × 3, and each type of image (RGB, HSV, and Mark) was divided into a training set, a verification set, and a test set in a ratio of 7:2:1.

### 4.2. Models Training and Testing

We set hyper-parameters k = 2, n = 50, and weight vector [0.5, 0.5], which means two classifiers (RGB and HSV) have the same contribution. Experiments were conducted in four ways and compared with each other: (a) training and testing RGB datasets only; (b) training and testing HSV datasets only; (c) training and testing both RGB and HSV datasets; and (d) training and testing other open-source methods with the RGB dataset, among which this paper chose DeepLab V3+ and SegNet.

**Figure 5:**
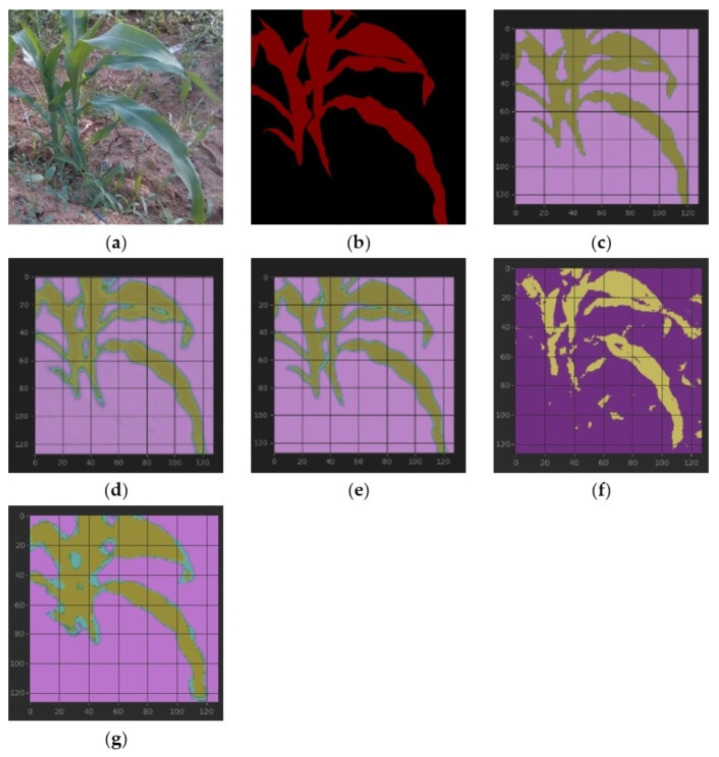
From (**left**) to (**right**) and from (**top**) to (**bottom**): (**a**) raw maize; (**b**) mask; (**c**–**e**) segmentation results of RGB, HSV, and our proposed framework with table lines as scale marks; and (**f**,**g**) segmentation results of DeepLab V3+ and SegNet. IoUs of (**c**–**g**): 0.8078, 0.7909, 0.8276, 0.6498, and 0.7313, respectively. (**e**): The blade corners are closer to the mask than (**c**,**d**). (**f**): There are many fragments. (**g**): Rougher. Both intuition and IoU show that (**e**) has the best segmentation effect.

Figure 5 shows the semantic segmentation results on our dataset Maize. Each type of training was executed for 50 epochs, a single UNet takes about 4 min, and the parameter size is 4.59 MB. Our ensemble framework doubles the training time and parameter size. Due to the small number of parameters of UNet, we set batch_size to 32, and the machine for algorithm execution was an MSI laptop. The main configuration of our hardware included an Intel(R) Core(TM) i7-8750H CPU @ 2.20GHz, 32.0 GB, NVIDIA GeForce RTX 2060 (6 GB) (Intel, Santa Clara, CA, USA). The parameter size of the network DeepLab V3+ is 139MB, and the batch size was set to 1 (a larger batch_size causes GPU memory allocation overflow, which needs to be further optimized). Finally, we obtained a total training time of 10.5 h. If the batch size is estimated to be 32, training 50 epochs also needs more than 20 min. Meanwhile, SegNet’s parameter size is about 112 MB, and training time is about 1.26 h under a batch size of 4.

**Figure 6:**
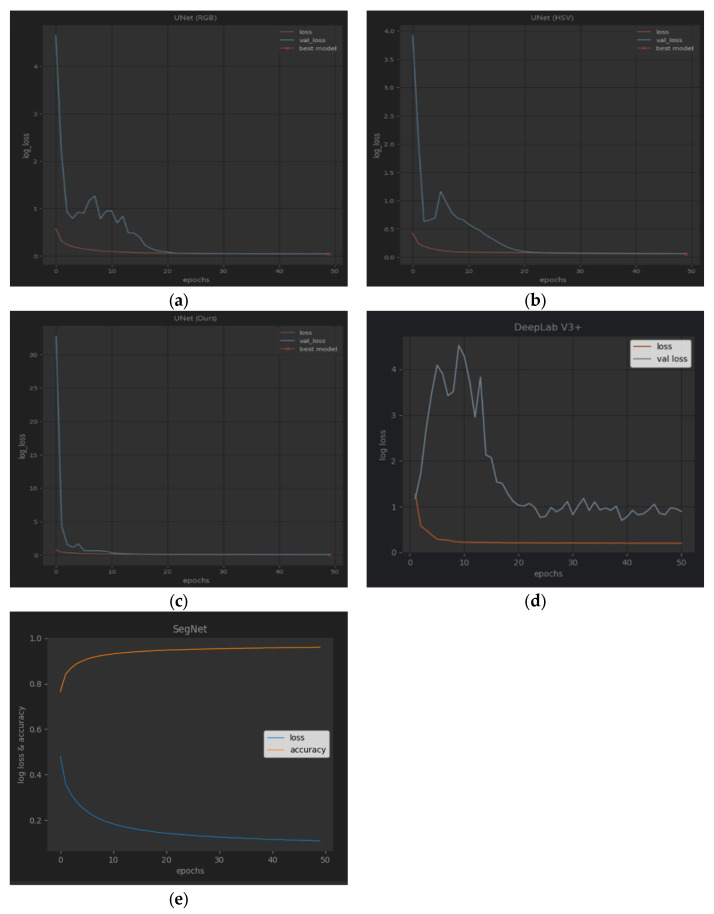
From (**left**) to (**right**) and from (**top**) to (**bottom**): (**a**–**d**) training and validation curves of RGB, HSV, our proposed framework, and DeepLab V3+; (**e**) SegNet’s training curves with loss and accuracy. The verification loss of (**d**) DeepLab V3+ fluctuates significantly at the beginning.

Figure 6a–d show the loss change curve of each type of training, including loss and val_loss. (e) is SegNet’s training curves with loss and accuracy. It can be seen from the curves that the verification loss of UNet (RGB) and UNet (HSV) fluctuates, but our framework is relatively flat. The verification loss of DeepLab V3+ fluctuates significantly at the beginning. The training and verification accuracy of a single UNet method can reach more than 0.85, and our ensemble framework can improve the accuracy by about 0.03 compared with a single UNet method. The training and verification accuracy of DeepLab V3+ can reach 0.97 and 0.89, respectively. It is observed that after the 25th epoch, the accuracy changes slightly, and the training can be terminated early to shorten the running time. SegNet’s training loss descends fast when beginning but slow in the end, and we obtain an accuracy of more than 0.96.

### 4.3. Assessing Results and Analyses

Ablation tests: In Figure 5, corn plants’ actual and predicted segmentation is compared. It can be observed that the values obtained in the RGB and HSV spaces are more or less similar in general, but there are also differences in some small areas, indicating that different color spaces have supplementary information. The ensemble framework adopted in this paper synthesizes each color space’s advantages, and the results obtained are better than single UNet methods. From the curve in Figure 6, it can be seen that the model is not severely overfitted. It is worth mentioning that some instabilities were observed during training, possibly due to the small number of samples in the dataset. At the same time, it is also noticed that in the boundary prediction, the framework of this paper overestimates a bit. This shortcoming may be avoided if a more extensive dataset is used to train the model.

Comparison with DeepLab V3+ and SegNet: Although DeepLab V3+ exceeds the UNet-based methods in terms of training and verification accuracy, from the segmentation results in Figure 5, DeepLab V3+ misjudged some small areas. From Figure 6, DeepLab V3+ has large fluctuations in the early stage, and the single UNet and our ensemble framework perform better. In addition, the UNet-based network has faster speed and smaller parameter space, which is more suitable for deployment in resource-constrained environments such as mobile robots, and our framework also has significant advantages in real-time performance. SegNet performs better than DeepLab V3+ but is inferior to UNet-based methods, except sometimes when it has a relatively better IoU than HSV. SegNet and DeepLab V3+ have parameters of a similar size, but SegNet is faster than DeepLab V3+ in training.

In contrast, UNet-based methods have smaller parameters and faster speeds. Table 1 illustrates all of this more clearly, and it is noteworthy that DeepLab V3+ obtained the best test accuracy but the worst IoU. From further testing, we found that the raw dataset size has a substantial impact on it, and an increase in the dataset can be considered to improve its IoU, but this goes against the original intention of using a small dataset only.

Improvements: (a) There are still human factors in determining hyper-parameters, such as the epoch size n and the weight vector [*ω*_1_, …, *ω_k_*]. In training and verification, dynamically adjusting hyper-parameters according to progress is a further step in the optimization direction. (b) In addition to HSV, there are other color spaces, such as YUV, and the integration of the model can be further optimized. (c) The research in this paper is a vital prerequisite for the environmental perception of agricultural robots. In addition to the requirements for computing speed and resource occupation, agricultural objects are complex, and how to study the rapid perception of new objects is a meaningful topic.

### 4.4. Sugar Beets Public Dataset

**Figure 7:**
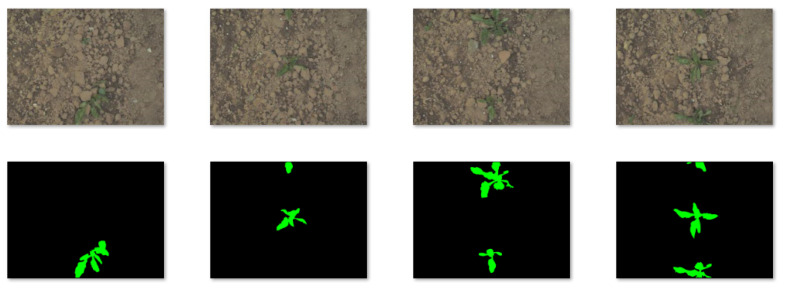
Sugar Beets Public Dataset. (**Top line**): images; (**bottom line**): masks.

To further verify the universality of our proposed framework, we tested it on a public dataset named Sugar Beets [27]. It presents a large-scale agricultural robot dataset for plant classification, localization, and mapping that covers the relevant growth stages for robotic intervention and weed control. Figure 7 shows several images and masks. Two things must be explained: (a) We only selected 30 images in Sugar Beets as the raw dataset and enhanced them, just like our self-built dataset Maize. (b) We only dealt with the binary classification problem, including sugar beets and environmental background (including weeds and ground), so we labeled weeds as background in the masks.

**Figure 8:**
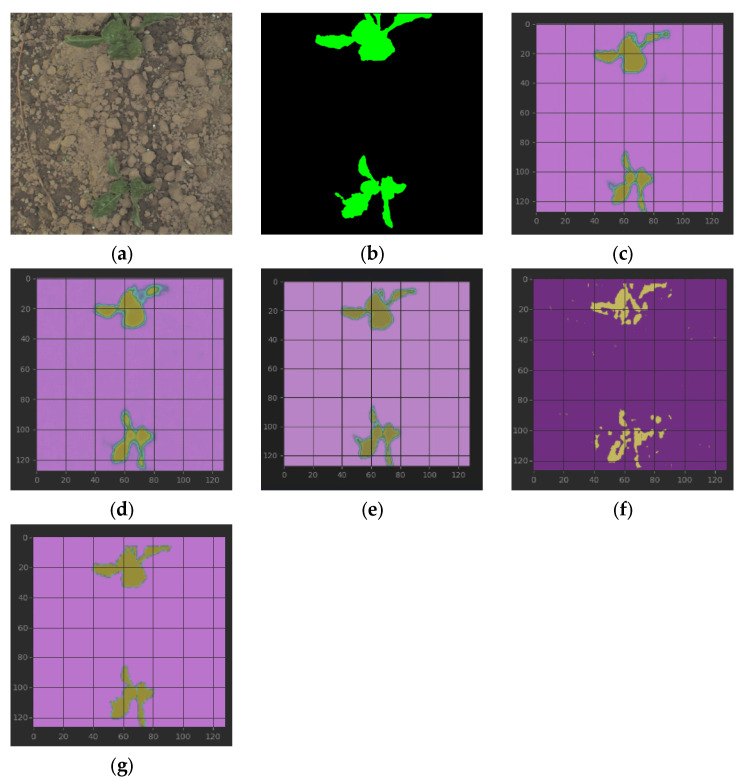
From (**left**) to (**right**) and from (**top**) to (**bottom**): (**a**) raw sugar beets; (**b**) mask; (**c**–**e**) segmentation results of RGB, HSV, and our proposed framework with table lines as scale marks; and (**f**,**g**) are segmentation results of DeepLab V3+ and SegNet. It can be seen intuitively that except DeepLab V3+, the segmentation effects of other methods are relatively good. IoUs of (**c**–**g**): 0.6848, 0.6513, 0.6972, 0.4112, and 0.6573, respectively.

Figure 8 gives the segmentation results of all discussed methods on Sugar Beets. We can find that methods based on UNet obtain relatively good results, while the IoU of (g) SegNet is a little better than (d) HSV. DeepLab V3+ is not good enough in this test and the preceding dataset Maize. Maybe the raw tiny dataset is the critical unfavorable factor. Refs. [28,29] also used the dataset Sugar Beets and obtained a better IoU than ours, but they used larger raw datasets, which were not precisely the same as our goals for the robotics occasions.

## 5. Conclusions

Semantic segmentation is of great significance in the application of agricultural intelligence, and it is an essential advancement for agricultural robots to perceive the environment and operate autonomously. This paper studies the semantic segmentation of crops in the field of agriculture. It proposes an ensemble framework based on the bagging strategy and UNet network, using two color spaces of RGB and HSV simultaneously and experimenting with a self-built dataset, Maize, and a public dataset, Sugar Beets. We compared our framework with UNet-based methods (single RGB and single HSV), DeepLab V3+, and SegNet. We discussed the results from three aspects: (a) data preprocessing, (b) model training and testing, and (c) assessing results and analyses. The evaluation results show that our framework has advantages in parameter size and execution speed, and it can be applied to agricultural robotics occasions.

We aim to study further how to upgrade the segmentation effect, execution efficiency, and intelligence by (a) self-searching and selecting hyper-parameters, such as the epoch size n and the weight vector [*ω*_1_, …, *ω_k_*]; (b) identifying and integrating more color spaces, such as YUV; and (c) automatically extracting more environmental information, such as depth of semantics segmented objects.

## Figures and Tables

**Table 1:** Performance comparison of all methods tested on self-built dataset Maize. Hardware: Intel(R) Core i7-8750H 2.20 GHz, Memory 32.0 GB, NVIDIA GeForce RTX 2060 (6 GB).

Method	Parameter Size	Training Time	IoU	Test Accuracy
UNet-RGB	4.59 MB	4 min	0.8078	0.8336
UNet-HSV	4.59 MB	4 min	0.7909	0.8274
Ours	9.18 MB	8 min	**0.8276**	0.8391
DeepLab V3+	139 MB	about 20 min	0.6498	**0.8512**
SegNet	112 MB	about 9.45 min	0.7313	0.8369

## Data Availability

The self-built dataset will be made available by the authors.

## Supplementary Materials

The Sugar Beets public dataset is available online at https://www.ipb.uni-bonn.de/data/sugarbeets2016/ (accessed on 7 September 2023).
